# Mississippi Valley Dental Society

**Published:** 1878-04

**Authors:** 


### MISSISSIPPI VALLEY DENTAL SOCIETY.

The thirty-second annual meeting of this body was held on the 6th, 7th and 8th of March. There was quite a large number present, and as a result a spirited and interesting time was had.

In the present number of the Register is given a report of a part of the discussions; anything like an adequate idea of the spirit of the meeting can not be obtained by reading the report; the social and professional animus that prevades such meetings can not be reported—can only be imagined by those who were absent.

This society, though surrounded by local and state societies, holds on in the even tenor of its way with about a uniform vigor and efficiency for good. Long may it live and flourish.

